# News and Miscellany

**Published:** 1882-01

**Authors:** 


### NEWS AND MISCELLANY.

Death of Four Horses from Eating Ensilage.—We have just been informed of the death of four horses which had been kept for about two months on a diet of ensilage. They belonged to a gentleman living in Woodstock, Vt. He began to feed them in November, 1881, giving about half a bushel of ensilage, with a little hay, three times a day. The animals died in January, with symptoms of dropsy.

The Number of Cattle reported in Texas for 1881, is 4,464,000 head, valued at $39,640,320.

The Number of Goats in Europe is stated to be 17,198,587, those in Denmark not being counted, as the goats there had not been reported when the census was made up. Greece possesses the largest number in proportion to her population—there being 1,339,538.

Tuberculosis has been found by M. Toussaint to become more powerful and rapid in its action the oftener it is inoculated.

Elephant "Bolivar," 9 feet 6 inches high, sold at the Van Amburgh sale for $7,100.

The Hog Products of last year in the United States aggregated 33,000,000, of which number 7,000,000 were handled at the Union stock yards, Chicago.

Fasting Dog.—A dog which had been accidentally confined at Metz, fasted 39 days, before he was released, and recovered.

M. Richenbach, in noticing the statement that Dr. E. C. Spitzka found in the egg of a turtle a live magot, says he once found in a hen's egg a small piece of printed paper.

Three Hundred Horses Burned.—The Fourth Avenue horse-car stables in New York City were destroyed by fire on the night of October 11th, and about 300 horses were burned to death.

Poison in Lupins.—It seems that there is a poison in lupins which produces in sheep a disease closely resembling jaundice. The virus can be neutralized, on the authority of Dr. G. Liebacher and Prof. Kuhn, by resorting to steaming.

Recipe for Sheep Dip Used in Texas.—
30 pounds of lime,
30 pounds of sulphur,
14 pounds concentrated lye,
15 gallons of water for the solution,
1 gallon solution to 12 gallons water.
The use of this dip is said in many cases to cure the scab, but it is questionable whether it will not damage the fibre and growth of wool. In this latitude tobacco dip has given the best satisfaction in all respects.

SHEEP SCAB IN AUSTRALIA.—It is said the Australians have a very stringent law for the eradication of scab in sheep. They have the State Scab Inspectors, whose business it is to see that the law is enforced. Every sheep owner who discovers indications of scab in his flock is obliged to notify all flock masters within a certain radius of the fact, and also to post notices in public places. If the disease is not stamped out within ninety days the diseased animals must be killed. The result has been that scab has almost disappeared from Australian flocks.

MARGOSSE OIL.—Sir Samuel Baker, in his "Rifle and Hound in Ceylon," mentions this oil as being a most valuable balsam for wounds, its peculiar smell, which is even more offensive than assafœtida, preventing the attacks of flies, which otherwise would blow the sore and occasion a nest of maggots in a few hours. It has also great healing properties, and soon creates a healthy appearance in a bad cut. It is manufactured from the fruit of a plant in Ceylon, but Sir Samuel Baker has never seen it in the possession of an English medical man.

M. PASTEUR has been elected a member of the French Academy.

THOMAS C. COWLEY, V.S., who graduated last March, died in St. Louis.

OIL CAKE must not be fed to cows alone. For cows that are dry, feed two pounds of oil cake with four to six pounds of corn meal per day. Avoid sameness in diet. Corn meal alone is likely to put the cow's system into a feverish condition.

CONSUL-GENERAL WEAVER writes from Vienna, Austria, to the Secretary of State at Washington, that rinderpest has broken out in an epidemic form in the province of Lower Austria, and has resulted in a loss from the disease, and by the destruction of animals—cattle, sheep and goats—supposed to be infected, of 3,088 head.

WHITE HORSES.—The horse has always been one of the most sacred animals among peoples of the Aryan race. Even in the old Hindu poems the sacrifice of a horse constitutes the most important ceremony in the primitive Aryan religion. The *Cornhill* reminds us that the Germans of the time of Tacitus kept White horses in their temple enclosures at the public expense, and took auguries from their snortings and neighings. The horse was buried by the dead warrior's side, and in military funerals still accompanies his master's body to the grave. But it was among the Low Dutch and early English tribes near the old mouths of the Rhine and Elbe that this animal was held especially sacred. A white horse rampant is still the cognizance of Hanover and Brunswick. When the Saxons, Angles and Jutes invaded England they brought with them their emblem of the white horse, which subsequently became the ensign of Kent, the first of the Anglo-Saxon kingdoms. The leaders of this first contingent of invaders, whether real or mythical, were Hengist, that is, stallion, and Horsa, that is, mare. Hence, too, the frequent occurrence of the word horse in places associated with the Anglo-Saxon conquest, as in the case of Horsham, Horsley, Horstead, Horstead Keynes, while the progress inland of the West Saxons would seem to be marked by the white horse cut in the chalk downs of Wantage and Westbury. The final victory of Egbert over the West Welch or Cornish was gained at Hengestesdun, that is Horsdown—now Hingston—in Cornwall. When, too, the missionaries first arrived in the island, the eating of horseflesh was made the test of adherence to English heathendom, while even in these matter-of-fact days a horse-shoe is by many regarded as a lucky object.—*Land and Water.*

One Cause of Colic in Horses.—Colic in horses is often brought on by feeding hay passed through the corn-stalk cutter, mixed with meal, middlings, or bran, and then wet up. The horse eats this food, thus prepared, so rapidly, that it is not properly masticated, and consequently becomes so clied in the stomach as to cause indigestion, followed by colic; more especially if directly after eating he is allowed to drink heartily of water; and the colder this is, so much the more liable to bring on colic. The best way, when a horse is brought into the stable, is to let him stand a short time, particularly if sweating, then give him three or four quarts of water, not over cold; then some uncut hay; after this a feed of grain or meal; and half an hour or so after that is eaten, all the water he pleases to drink. Some horses will eat cut hay with impunity, others cannot, or at least not till after they have first eaten some uncut.—*Live Stock Journal.*

A New Veterinary School.—In connection with the Minnesota College Hospital there is to be attached a veterinary school, where students will receive a full course of instruction in the different branches of veterinary medicine and surgery, extending over three winter sessions, of six months each. The faculty are to consist of Richard Price, V.S., F. W. McLellan, V.S., of St. Paul; H. J. Burnash, M.D., S.R.C.P., and C. C. Lyford, M.D., V.S.

An English Buyer of Horses in the United States.—A Mr. Fowler, the agent of a large omnibus company in England, is now among us for the purpose of making purchases. We should think the half-bred horses got by the large class of stallions, like the Percheron-Normans, Clydesdales, and others, out of common mares, would suit him best for this purpose, if they have good, tough, sound feet and legs. These are very essential, as the horses are to be used on paved streets and hard macadam roads entirely. None but the best of feet can stand the wear and tear of such, and, in fact, they are very important even for softer country roads, as the grit and gravel which abounds more or less in them, are quite wearing to the hoof; and then weak legs are more likely to be sprained on these than on harder roads, as, on account of the mud holes, extra hard pulling is often required. —*Live Stock Journal.*

Public Health Association.—The ninth annual session of the American Public Health Association met at Savannah, Ga., on the 29th of November. There was a large attendance. The meeting was called to order by the President, Dr. Charles B. White. The following papers were read at the morning session: "The Contagious Diseases of Domestic Animals," by Dr. Ezra M. Hunt, of New Jersey; "Diseases among Texas Cattle," a continuation of the report made to the association at the New Orleans meeting, December, 1880, by Dr. J. R. Smith, United States Army; "A Report of the Examination of Hogs at the New Orleans Abattoir During the Summer of 1881," submitted by the New Orleans Sanitary Auxiliary Association; "Trichinæ Spiralis," by Dr. J. M. Partridge, of Indiana; "Trichinæ Spiralis in American and German Hogs," by Dr. F. S. Billings, veterinary surgeon, of Massachusetts.

Horses Finding Their Way Home.—When going down to Melbourne last year, I left my two horses at a station 300 miles from here. I got them the day I got back there, but the next morning they could not be found, nor could they be found after two days' hunting. I made up my mind they had been stolen out of the paddock. I therefore bought two others to bring me on here (Currawella). I was much surprised, a few weeks ago, to find they had come back the 300 miles themselves, and were running with their own mob on the run. I can any time lend a horse to be taken away fifty miles, and if he is let go he will be back in a couple of days. I once had two horses get away from me in the night, and, although close-hobbled, they went forty-five miles before overtaken.—*Field.*

The Consumption of Horseflesh in Germany is growing. A very careful supervision is exercised over the trade in Berlin. The inspector has a list of the stables where the existence of any contagious disease has been reported, and if he finds that the animal brought before him comes from any of these, a prosecution against the seller is at once instituted. Should the horse be found by the veterinary surgeons to be suffering from any disease not contagious, it is at once killed; but the body is sent to the Zoölogical Gardens. The Berlin butcher pays about $10.50 for a piece of horseflesh weighing from 250 to 300 pounds, but he retails it at 10 cents a pound for the filet, 6¼ cents per pound for the other pieces, and 5 cents for parts only fit to be made into sausages; and as horseflesh is naturally very dry, a good deal of it can be only utilized by being mixed with lard and converted into sausages, which, it may be added, are, it is shrewdly suspected, largely consumed by persons who are little aware of what they are eating. In one or two other German towns the consumption of horseflesh is, in proportion to their population, even larger than in Berlin. In Breslau, for instance, a town with 250,000 inhabitants, 2,000 horses are killed annually for the market; and in Altona, with a population of 100,000, the number reaches 1,500. In the western provinces, on the other hand, horseflesh is more rarely eaten even in the more densely peopled towns—the average number of horses killed annually in Dortmund being only 240, and in Bielefield about 100.—*World.*

Advance in the Price of Horses.—The Boston *Advertiser* says that there is a sharp demand for good horses at an advance of from fifteen to thirty per cent. on the values current the past two years. In fact, at no time within twenty years has there been so noticeable a scarcity of thoroughly sound stock. The horse car companies, that three years ago were supplying their wants at from $82 to $100, are now forced to bid up to $130 for animals counterparts of their 1879 purchases. Truckmen, who were last autumn buying heavy draught teams at $350 to $400, are now compelled to pay $425 to $550 for duplicates for these pairs. The most persistent demand is for dark-colored, well-bred carriage horses, in closely-matched pairs, each horse weighing about twelve to thirteen hundred pounds. Such a well-broken and stylish team, though not fleet, will secure from $600 to a $1,000, the price advancing from the lower range upon their freedom from blemish and their evenness in speeding. The major part of the receipts of horses are from Ohio.

Japanese Food.—M. T. Van Buren, our Consul-General at Japan, presents in a Blue Book some interesting facts in regard to the food of the Japanese people. With a population of 30,000,000, there is to be found in the whole country but little more than 1,000,000 head of cattle. Of these only 600,000 can be considered as fit for food. Therefore there are but two head of cattle for each 100 people, whereas in the United States we have for 100 mouths 73 cattle to fill them. Japan slaughters, however, 36,000 head of cattle, more than one-half of which is eaten by the foreign population, the rest being consumed by the Japanese navy and army. Mutton and pork are, outside of the treaty ports, almost unknown. Fish enters largely into the food of the people. Mr. Van Buren mentions that "cod, salmon, herring, mackerel, salmon trout, carp, eels, skate, mullet, cat-fish, and plaice are plentiful and cheap." It is known that the Government has taken active measures in regard to fish-culture, and endeavors in every way to increase the products of the sea, sending for all American publications on these topics. The Consul states that "one-half of the people eat fish every day, one-quarter two or three times a week, and the balance perhaps once or twice a month." It is their habit to eat a great many varieties of fish raw. But the Japanese are more essentially vegetarians than even the Chinese, and all the land and marine plants, with the tubers, seem to be placed under contribution. Among exceptional food plants, Mr. Van Buren mentions an acorn which grows on a small bush from three to four feet high, "it has less sugar than the nut from the chestnut tree of America, but has the merit of being free from astringent and bitter qualities. Large quantities of these nuts are gathered, dried, and eaten by the people in various ways." This edible acorn would be worthy of introduction into this country.

Influences of Parents.—Mr. Howard lays down the following as "cardinal points" in the art of breeding:

1. That from the male parent are mainly derived the external structure, configuration and outward characteristics, the locomotive peculiarities inclusive.

2. From the female parent are derived the internal structure, the vital organs and, in a much greater proportion than from the male, the constitution, temper and habits.

3. That the purer the race of the parent, the more certainty there is of its transmitting its qualities to the offspring. Say two animals are mated; if one is of purer descent than the other, he or she will exercise the most influence in stamping the character of the progeny, particularly if the greater purity is on the side of the male.

4. That, *apart from certain disturbing influences or causes*, the male, if of pure race and descended from a stock of uniform color, stamps the color of the offspring.

5. That the influence of the first male is not unfrequently protracted beyond the birth of the offspring of which he is the parent, and his mark is left upon subsequent progeny.

6. That the transmission of diseases of the vital organs is more certain if on the side of the female, and diseases of the joints if on the side of the male parent.—*Country Gentleman.*

Cattle Restaurants.—Mr. Alfred D. Tingley, of the Humane Live Stock Express Company, 2 Wall street, has invented a scheme which he thinks will put a stop to the present inhuman system of sending cattle long distances without food or water, and slaughtering them while in the unfit condition caused by this treatment. Formerly he invented a feed car, which was tried, but was not a success. The grain and water were placed on the roof, and passed down by pipes when required; but the troughs in the crowded cattle cars got dirty, and the animals refused to eat out of them. An attempt was then made to substitute cars with compartments, so as to keep the cattle separate, but this rendered the cars unfit for any other purpose on the return trip, and was abandoned.

Mr. Tingley's present scheme is a simple one. It is to establish a number of "cattle restaurants" along each line of railroad that transports live stock. They will be 200 miles apart, and the cattle can be fed and watered every twelve hours. When a train with a load of cattle on board gets within twenty miles of one of these restaurants a telegram will be sent to the officer in charge, and when the train arrives everything will be in readiness. Great iron cups, about as large as, and something of the shape of, a good-sized kitchen-pot, will contain food and water, run into them through rubber pipes from tanks above. The train will stop between two rows of these troughs, those on one side containing water, and those on the other side holding four quarts of food, consisting of a mixture of ground corn, oats and cut hay. Each car will have sixteen openings on each side, all of which can be easily closed when the car, which need be nothing more than an ordinary cattle car, such as is at present used, is required for other purposes on the return trip. Into each of these openings a trough with food or water will be pushed by means of a sliding bar upon which it rests. It will move forward to the car direct, or sideways, as may be required to reach the opening, the side motion being accomplished by sliding it along another bar, extending the whole length of the restaurant, the bar by which it is pushed forward accompanying. The flexible rubber tubes through which the food and water passes will, of course, offer no resistance. Mr. Tingley has in his office a model of a restaurant.—*Sun.*

The Claims of Comparative Pathology.—Veterinary Surgery has recently assumed a much more dignified position in the popular regard, and in the opinion of the medical profession. It has medical journals, some of which are creditable in spirit and contents, and in one or two of the medical schools it seems to be looming up as a branch of some importance in the future. It is rumored that a veterinary college is about to be started in St. Louis, and another under the auspices of an old established medical institution in Philadelphia. A course of lectures on Comparative Pathology is also to be delivered before the medical department of Yale College. A good education in this direction seems to be a great desideratum, for quackery in the field of veterinary science has been too long an unchecked evil, for which we presume no absolute remedy has yet been offered. While the tendency of the times is to expose illegitimate medical practice, and to restrain the abuses which have hitherto characterized it, little or nothing has been done, legally, for the protection of domestic animals, and in this protection to preserve the valuable property of many thousands of the people. There are many pretenders in the ranks of these practitioners, who have no claim whatever to be considered veterinary surgeons, and the sooner they are exposed the better for the reputation of all existing members of that fraternity who have been properly trained and educated in the study of the anatomy, pathology and therapeutics of the lower animals. A veterinary surgeon, as self-styled, in Chicago, recently ordered four ounces of tincture of aconite root from a pharmacist, whose curiosity led him to ascertain the fact that the prescriber believed that tincture of aconite root and sweet spirits of nitre were one and the same thing. If examination and graduation in this branch were obligatory on all those who intended to practice it, such ignorance would be scarcely possible.—*College and Clinical Record.*

The Practical Value of Vivisection.—Dr. Urban Pritchard writes to the London *Times*, under date of November 4:

"I see by the *Times* of to-day that a summons has been granted against my friend and colleague, Prof. Ferrier, for certain experiments on animals which were undertaken for the advancement of our knowledge of the functions of the brain.

"I do not wish to discuss the merits of the case, which, no doubt, will be carefully considered and impartially dealt with; but I am anxious to add my testimony, however insignificant, to the practical value of Dr. Ferrier's work in my limited branch of medicine. On many occasions I have been considerably assisted both in diagnosis and treatment of nerve deafness by Prof. Ferrier's knowledge gained in the course of his experimental studies.

"Of course his work is, and will be, of far greater value to the general physician. I am confident that the medical profession at large feel so strongly on this point that Dr. Ferrier will not be allowed to bear any pecuniary loss should this case, through some technical omission on his part, be decided against him.

"I cannot help lamenting the harm that is being done to the advancement of the healing art by false sentiment fostered by many real well-meaning persons. Is it not sad to think that a professor should be driven to the Continent to perform experiments which have for their object the perfecting of a system which has already saved thousands of lives? And yet it is a fact that such did occur last year to another eminent London professor."

A Wonderful Bird.—There is now in the London Zoölogical Gardens a remarkable bird, the Nestor Notabilis, or mountain Kea, of New Zealand. It is a parrot of strong frame and powerful bill and claws, which were used like those of all parrots for obtaining a vegetable diet, until the colonists introduced sheep and pigs. As soon as this was done the Kea seems to have abandoned vegetable food, and to have taken entirely to flesh eating. He attacks sick or disabled sheep, and with his powerful cutting beak opens a passage through the back, and eats the intestines. Even healthy animals are sometimes assailed by the Nestor Notabilis, and there are sheep runs in New Zealand where considerable losses have been incurred through these strangely degenerated birds. The specimen in the Zoölogical Gardens gave as much trouble to capture as an eagle, tearing the clothes of the shepherd who knocked it down while pouncing on a lamb, and lacerating his hands. The Kea scorns cooked meat, biscuits, fruit or seeds, and likes raw mutton better than any food. He will tear the skin and flesh from a sheep's head after the furious fashion of a vulture, leaving nothing but the bare skull. He at one time holds the morsels in his lifted claw, after the style of parrots, and at another grips them under his feet while rending with his beak like a hawk.

Meat from Cattle Suffering from Splenic Fever.—At Birkenhead Police Court on October 6th a wholesale butcher of Liverpool was fined £5 and costs for having exposed for sale the carcase of a bullock which had been condemned as unfit for human food. The animal was one of a cargo of cattle from Boston, some of which died on the voyage from splenic fever. The inspector found the visceral evidences of this disease in this case, and condemned the meat, but it was conveyed to Liverpool and there sold. The evidence given was of a most conflicting character. The Birkenhead authorities, including Dr. Vacher, the medical officer of health, deposed to the flesh of animals dying of splenic fever being unfit for food; but the Liverpool inspectors and a number of butchers gave contrary evidence, many of the latter stating that the beef was "splendid," whilst Mr. Luya, the chief meat inspector, asserted, in cross-examination, that "he had only condemned one beast for splenic fever. He had seen several cases, but the meat was sold. Splenic fever came from America. As a usual thing he would not condemn a beast for splenic fever unless it was a very bad case. He did not think the meat of a beast which suffered from splenic fever was injurious to health." We are glad the magistrate was not to be dissuaded by such evidence from deciding against the salesman, for although "thorough cooking" *may* suffice to render the meat inocuous, the risks of inoculation during the dressing of the meat are quite sufficient to justify its condemnation. *London Lancet.*

Australian Wild Horses.—It is claimed for the enormous island continent which lies at the Antipodes that ere long it will be the greatest horse-producing centre in the world. The Australian colonies already possess a larger number of horses in proportion to human beings than any other country in existence; and so favorable is the climate and pasturage for raising live stock of all kinds that it will not surprise us if, following the example of the United States, Australia should send us some great race-horses to run for our best English stakes, and perhaps to win the Derby, before many years have passed.

Speaking summarily, there are about 1,000,000 tame horses in the Australian colonies, to which must be added a ragged lot of about 120,000 wild horses, which hang upon the skirts of civilization, and are as great a nuisance to the squatters as rabbits to the agricultural community. The wild horses, or "brumbies," as they are called, owe their existence to a few tame animals which have from time to time escaped from man's control and taken shelter in the bush, where they have multiplied with inconceivable rapidity. In his "Physical History of the Human Race," Dr. Prichard tells us that the most refined, cultivated and polished of men—a Sir Charles Grandison, for instance—would utterly lose his civilization and revert to a barbaric type, if confined for a year upon an island solely inhabited by savages. Something of the same kind takes place where a thoroughbred entire horse escapes into the Australian bush. He cannot lose his fine symmetry, his high courage and eye of fire, or his grand action; but his coat soon becomes as coarse and rough as that of the wild colts and mares by which he is surrounded, and his progeny conform to the straight-shouldered, cat-hammed, and spindle-shanked type of their villanous dams. But such is the inborn partiality of the noble animal for liberty, that there is no instance known in which a runaway from civilization has returned voluntarily to slavery, although it might be imagined that man's care is more than sufficient compensation to a horse for the reasonable amount of work exacted from him. Nor, to all appearances, is wild life in the bush so attractive as to justify the preference shown for it by horses which have once served man as domestic animals. The pasturage is often very scarce, and in years of drought the rivers, brooks and water holes dry up, so that the "brumbies" have to travel hundreds of miles to get food and drink. Man's hand is against them wherever they go, and they end by becoming as unapproachable as the mountain sheep of North America, or the wild ass of Africa. In one respect they do not altogether lose their domestic instincts; for it is their habit to hover continually on the outskirts of civilization, partly from a desire to tempt tame animals to follow their runaway example, and partly because they know that where man dwells forage will not be wanting.—*London Field.*

Exports and Imports from the Port of New York Last Year.—The exports of live stock and fresh meats from this port during the year ending Nov. 30, 1881, aggregate in value $10,533,809, more than one-half of which sum is represented by fresh beef. Horned cattle, 39,968 in number, come next in point of value, being worth $3,934,865—nearly $100 each.

We sent away 26,441 sheep, valued at $317,794; 873 horses, worth $235,190; 1,668 mules, valued at $201,160, and 3,782 hogs, valued at $44,002; 1,706,164 pounds of fresh mutton, worth $132,287. The quantity of fresh beef exported was 60,211,653 pounds, and its value was $5,688,511. In live cattle, the falling off, as compared with the year ending Nov. 30, 1880, was 45 per cent., or from a value of $7,411,904 to $3,934,865. In fresh beef the reduction is from $6,352,096 to $5,688,511; in hogs from $82,831 to $44,000; in sheep from $367,315 to $317,794, and in mutton from $183,542 to $132,287. In all these items together—live cattle, sheep and hogs, fresh beef and mutton—the falling off in exports from this port alone is $4,280,129. It is to be noted that the decline in British receipts of living animals for the eleven months ending Nov. 30, was £1,673,000, showing that the falling off in importations from the United States was not made up from other countries.

The following table shows the importations of animals at this port for 1880 and 1881. These animals were chiefly thorough-bred horses, Norman draught horses, and Shetland ponies, Channel Island cattle, herds from Holstein, and me lium wooled sheep from the British Isles:

| FOR 1880. | No. | Value. |
|---|---|---|
| For breeding, all kinds | 1,174 | $325,792 |
| Horses paying 20 per cent. duty | 37 | 9,445 |
| FOR 1881. | | |
| For breeding— | | |
| Horses and ponies | 919 | $378,133 |
| Jacks | 10 | 3,895 |
| Cattle | 442 | 92,669 |
| Sheep | 300 | 6,867 |
| Horses paying 20 per cent. duty | 19 | 1,559 |
| For breeding, all kinds | 1 671 | 481,564 |

Values all per invoices.

—*New York Times.*

THE HIPPOPOTAMUS.—Dr. Henry C. Chapman, of Philadelphia, has recently devoted much attention to the anatomy of the hippopotamus, and has read an elaborate paper before the Academy of Natural Sciences of Philadelphia. We notice that he draws the following conclusions: "Beginning with the pig, we pass by an easy transition to the peccary, which leads to the hippopotamus, and thence in diversing lines to the ruminantia on the one hand and the manatee on the other. Paleontologists have not discovered a form which bridges over the gap between the hippopotamus and the manatee, but it will be remembered that certain ferril bones, considered by Cuvier to have belonged to an extinct species of hippopotamus, *H. medius*, are regarded by Gervais as the remains of the *Halithorerium fossile*, an extinct Sirenean, of which order the manatee is a living representative." Dr. Chapman adds further on, "I don't mean to imply that the manatee has necessarily descended from the hippopotamus," but he considers that "there is some generic connection between them."—*Science.*

JIBBING HORSES.—A correspondent, writing to the *Times*, propounds a new theory that jibbing is the result of "a temporary mental state" and that "the object in view should be to divert the small mind of the horse from its one prevailing idea." The writer proceeds to suggest methods of diversion, which will be diverting to the readers if not to the horse. For example, he recommends the drivers of "jibbing horses" to be provided with a small bottle of something that a horse abhors, and which stings or irritates. By placing a small portion on the tongue of the horse all its thought is directed to clean its mouth, and every other consideration is entirely from its mind. We are incontinently reminded of the storied way of catching birds by sprinkling salt on their tails. No doubt the remedy would be successful. Meanwhile, there is nothing whatever new in the notion of jibbing being a mental vice—so is shying and so are certain forms of crib biting. That horses have temper and may become "moodish" or "modish," or savagely insane has been known to all students of comparative psychology and to most "vets" for a great length of time. Rarey recalled public attention to the matter a few years ago, and it was then clearly recognized by all intelligent horse trainers. Still better than the expedient recommended is to blow or whisper in the ear of a horse on the side opposite to that on which he inclines to kick—it is useless on the same side. If he either likes or dislikes the sensation enough to have his mind changed, he will go on. Nothing, however, can succeed in a case of *genuine* "jibbing," except it overcomes and cures the ill temper. In nine cases out of ten the jibbing is not genuine. The horse stops because the hames hurt him somewhere, and he is sulkily resolved not to proceed until the grievance is redressed. When there is no grievance the horse is like a perverse child, and "giving him something he does not like" is a form of punishment which diverts his small mind, just as the small mind of a child is diverted by a good whipping. We do not advocate whipping, but neither can we recommend coaxing. The best plan is to look for a cause of irritation, and to change the mind state, as we have indicated.—*London Lancet.*

